# Determining staffing standards for primary care services using workload indicators of staffing needs in the Philippines

**DOI:** 10.1186/s12960-021-00670-4

**Published:** 2022-01-28

**Authors:** Ma Graziella Aytona, Mary Ruth Politico, Leah McManus, Kenneth Ronquillo, Mollent Okech

**Affiliations:** 1 Department of Health, Health Human Resources and Development Bureau, Manila, Philippines; 2 Chemonics International, Washington, DC United States of America; 3 World Health Organization, Port Moresby, Papua New Guinea

**Keywords:** Primary health care, Universal health care, Workload indicators of staffing need, Health workforce, Health workforce planning, Staffing requirements

## Abstract

**Background:**

Health services cannot be delivered without an adequate, competent health workforce. Evidence suggests a direct relationship between density of health workforce and health outcomes. The Philippines is faced with health workforce challenges including shortages, inequitable distribution and inadequate skill mix which hinder health service delivery. Evidence-based workforce planning is, therefore, critical to achieve universal health care.

**Methods:**

The Philippines adopted the World Health Organization’s workload indicators of staffing need methodology. Using a multistage sampling method, nine regions with poor health indicators in tuberculosis, family planning, and maternal child health were identified. Physicians, nurses, midwives, and medical technologists were prioritized in the study from 89 primary care health facilities (barangay health stations, rural health units, and city health offices). Data was collected using in-depth interviews, document reviews, observations, and field visits. The workload indicators of staffing need software were used for data analysis to determine staffing requirements and analyse workforce pressure.

**Results:**

The study showed varied results in terms of staffing requirements and workload pressure across cadres and facility types. Some health facilities exhibited staff shortages and high workload pressure. Out of the 40 rural health units and city health offices, only three had the required physicians needed and 22 facilities had a shortage of physicians working under high workload pressure. Other facilities had excess staff compared to the calculated requirements. Nurses at the rural health units showed high workload pressure. Ten rural health units had no medical technologists. Midwives at barangay health stations exhibited extremely low workload pressures.

**Conclusion:**

The study identifies the need for the Philippine Health System, both through the Department of Health and the local governments to efficiently optimize the available health workers by revising the services offered at the primary health care facilities. The results provide evidence for staffing requirements at various levels of care based on workloads, scope of practice and time taken to undertake specific tasks at the barangay health stations, rural health units and city health offices to be integrated into the human resources for health management systems.

## Background

The 1978 Declaration of Alma Ata on primary health care (PHC) revolutionized the world’s interpretation of health with the core principles of universal access to care, equity, community participation, intersectoral collaboration and appropriate use of resources [1]. Moving forward, reforms towards Universal Health Coverage are hinged on strong primary care systems to provide essential health services to all.

The Philippines has a long history of PHC having adopted the approach in 1981 as a national strategy. This strategy relies heavily on the community through barangay health stations (BHS) that serve a population of 5,000 and rural health units (RHUs)/city health offices (CHOs) that serve a population of 20,000 [2]. The devolution of health services in 1991 mandated the management of primary care facilities at the barangay, city, or municipal levels to local governments units (LGU) [3]. The DOH, on the other hand, sets the standards for primary care facilities, including their staffing. In addition to formal cadres of health workers under the primary care facility (e.g., physicians, nurses, and midwives), Barangay Health Worker (BHW) complement health services at the community level, acting as the first point of contact between the healthcare system and the rest of the community [4].

In 2019, building upon successes in the past 30 years of health reforms, the Government of the Philippines signed the Universal Health Care (UHC) Law (Republic Act 11223) which provides a strong agenda for effective health workforce management in the country [5]. The UHC Law highlights the importance of the primary care approach and provides for the formulation and implementation of human resources for health (HRH) policies and plans that generate, recruit, retrain, regulate, retain, and reassess the health workforce based on population health needs [5]. UHC ensures that everyone has access to well-trained, culturally sensitive, and competent health workers. The best strategy for achieving this is by strengthening multidisciplinary teams at the primary health care level [6–8]. Key in this endeavour is the availability of competent and well-motivated health workers at the community level [9]. The Philippines, however, faces several HRH challenges. These challenges include a shortage of health workers, maldistribution, and an urban bias that causes most rural areas to be severely understaffed. Some health workers are employed on a contractual basis, either by the government or development partners. This has negative consequences on retention and biases service provision towards specific disease programs [10].

The HRH shortages and inequities in the Philippines translate to disparities in the provision of quality of health care services, impacting critical PHC services, such as Tuberculosis (TB) and family planning (FP) [11]. TB remains one of the leading causes of morbidity and mortality despite sustained investments on the prevention, control and management by the government and partners. In 2016, the World Health Organization (WHO) reported that there were 260,000 projected cases in the country with 28,000 dying per year [11]. The report further highlighted the emergence of multidrug-resistant TB and extensively drug-resistant TB across population groups have significantly increased. In addition, the 2017 Philippines National Demographic and Health Survey indicated the low uptake of FP services noting that one in every five married Filipinas wishing to postpone their next birth or stop childbearing are not using contraceptive [12]. This is despite provisions in the Responsible Parenthood and Reproductive Health Law (Republic Act No. 10354) guaranteeing universal access to FP information in all public health facilities with emphasis in the primary care level facilities [13].

The UHC Law echoes the need for evidence-based planning for HRH at all levels of care with an emphasis on primary care. Evidence-based HRH planning provides the information necessary for mobilizing adequate resources based on these needs. Furthermore, it recognizes that having adequate staffing in health facilities requires critical consideration for HRH planning beyond the usual workforce to population ratios. [10, 14–16].

In response to this need to conduct evidence-based planning, the Philippine Department of Health (DOH), with support from the United States Agency for International Development (USAID) funded Human Resources for Health 2030 (HRH2030) Philippines Project implemented by Chemonics International, in 2019 used the World Health Organization’s (WHO) Workload Indicators of Staffing Need (WISN) methodology with a focus on the four most prevalent cadres, namely, nurses, midwives, physicians and medical technologists in primary care health facilities in selected regions of the country. While the DOH and other stakeholder conducted workforce analysis studies in the past using population and health worker densities, this study was the first in the Philippines to adopt the WISN methodology step by step to provide evidence for staffing requirements for the country’s context. The WISN methodology offers an objective and scientific method to estimate health workforce requirements based on actual workloads, looking at both the health service and non-health service activities that are conducted by health workers using actual service statistics from the facility [17–21]. The WISN study allowed the DOH to conduct a thorough analysis of the workload of physicians, nurses, midwives, and medical technologists at BHS, RHUs/CHOs. The study resulted in the identification of staffing needs, as well as minimum and maximum staffing standards, for these cadres to carry out PHC and ultimately contribute to achieving UHC.

## Methods

### Study design

The DOH and HRH2030 Philippines used a cross sectional study design to adopt the WISN methodology. The study included WISN variables, namely, the available working time, workload components (health services, support, and additional activities), service standards and the standard workloads for health service activities. Annual statistics for the year 2018 were used together with the existing staff for the four main cadres of focus in the selected health facilities.

### Setting

The study was set in the Philippines in the Luzon, Visayas and the Mindanao regions. Data collection was conducted from October 2018 to April 2019, while data analysis and report development were completed from April to July 2019. The study focused on services delivered by physicians, nurses, midwives, and medical technologists at BHS (49) and the RHUs/CHOs (40) that are managed by LGUs.

### Working groups

Three critical committees with specific roles to ensure accurate implementation, oversight and acceptable workload components and activity standards were established. The Steering Committee consisted of senior health managers and policy makers, chaired by the Undersecretary providing overall oversight and supervision for implementation. A national level technical task force (TTF) and three regional TTFs from the regions of focus were established. The national TTF consisted of representatives from the Health Human Resources Development Bureau (HHRDB) and other key offices within the DOH, selected key health workers from the four cadres and technical officers from the HRH2030 Philippines team. The national TTF was trained to ensure that they acquired skills and knowledge on how to use the WISN tool manually and electronically. The national TTF subsequently trained the regional TTFs. As the critical team to logically ensure implementation of the WISN methodology was ingrained in the country, the members of the TTF undertook an oath of commitment to ensure WISN was implemented logically and successfully as per the WHO guidelines. Finally, the last group formed was the expert working groups (EWG). The four EWGs consisted of experienced health workers from the four cadres of priority at all levels of care from both public and private health facilities. They underwent 3-day training on the WISN methodology. Their role was to establish realistic, reliable, and acceptable comprehensive workload components and activity standards based on actual and acceptable professional standards. To validate the workload components and activity standards developed by the EWG, a separate group made of up of the four cadres was formed to validate the workload components and it included representatives from the regulatory councils, professional associations and health training institutions.

### Sampling design, size, and procedure

Using a multistage sampling method, nine regions of the Philippines were identified with poor health indicators in maternal child health, FP and TB as reported by 2017 health statistics. The regions cut across the urban, rural, and geographically isolated and disadvantaged areas (GIDA) with a proportional representation from the three major island groups of Luzon, Visayas, and Mindanao. In the second phase of selection, DOH regional offices provided a list of facilities, where 49 BHSs and 40 RHUs/CHOs were randomly selected from to form part of the study.

### Data collection and analysis

Considering that the existing health information system did not gather all the data as established by the EWG during the development of the workload components, there was a deliberate need to collect specific data from the health facilities. A team of trained data collectors which included some members of the EWG and national and regional TTF visited the facilities and collected data using pre-developed Excel sheets to gather monthly health services statistics for the year 2018. Before data collection, the data collection team paid courtesy calls to the Provincial Health Offices. The offices served as the entry points to the health facilities and provided preliminary information on the health sector and staff records in the province. The Human Resources Officers provided information on the staff establishments, authorized and unauthorized leaves and actual hours worked per day and days taken for training or other reasons. They also shared other health systems issues that were relevant to the WISN process. Data for all the three workload groups were collected for the specific cadres in the various service areas in the health facility. The raw data was entered in the master Excel sheet, validated by team leaders, and finally uploaded into the WISN software to produce reports per facility and by cadre in web archive transformation files for further analysis.

### Limitations

There were some limitations to the study which included the sample size selection considering the overall number of facilities in the country and funding restrictions that prescribed the scope. In addition, there were gaps in data availability in the health facilities, while some data were aggregated for annual service statistics conducted by different cadres. These were mitigated by triangulating data sources, further interviews, and expert opinion.

## Results

### Trends in available working time

The study first calculated the available working time (AWT) for each of the cadres in the various facilities. AWT can differ from one facility to the other. Table 1 shows an example of an RHU AWT for four cadres. The nurse has 1744 h in a year, 1856 h for a medical technologist, 1864 for a physician and 1952 h for the midwife being the highest. In this facility, the nurse was out of the workplace for training for 17 days, while the other cadres took less than 5 days out of work due to training.

**Table 1:** Available working time for four cadres in a Rural Health Unit

Cadre	Working Days per Week	Working Hours per Day	Annual Leave	Public Holidays	Sick Leave	Special/No Notice Leave	Training Days	AWT in Weeks	AWT in Days	AWT in Hours
Physicians	5	8	8	13	3	0	3	46.6	233	1864
Nurse	5	8	8	13	4	0	17	43.6	218	1744
Midwives	5	8	2	13	0	0	1	48.8	244	1952
Medical Technologist	5	8	8	13	5	0	2	46.4	232	1856

### Workload group and activity standards per cadre

The study provides results based on the three workloads groups for physicians, nurses, midwives and medical technologists providing PHC services at the BHSs and RHUs/CHOs in the Philippines context. Tables 2, 3, 4 and 5 annexed provide the workload components for health services, support and additional activities. In addition, included in the tables are the activity standards and allowance factors for each of the cadres. For example, a workflow in an RHU/CHO is provided. A patient visits an RHU/CHO with symptoms of dengue fever; the nurse spends 13 min assessing the patient by welcoming, registering, taking vital signs and taking history before sending the patient over to the physician for examination. The physician in turn examines the patient during consultation that takes 16 min before ordering a confirmatory test to be conducted by a medical technologist in the laboratory. In the laboratory, the medical technologist spends 10 min conducting a dengue rapid test and interprets the results before sending the patient back to the doctor for prescription which is finally administered by the nurse. This is an indication of how the skill mix supports the health interventions.

**Table 2:** Workload components and activity standards for medical technologists

Workload Group 1: Health Service Activities	Activity standard
Complete blood count—automated	9 min/sample
Complete blood count—manual	25 min/sample
ABO and Rh blood typing	10 min/sample
Fasting blood sugar	33 min/sample
Oral glucose tolerance test (OGTT)	123 min/sample
Cholesterol	9 min/sample
Creatinine (serum/urine)	9 min/sample
Lipid profile	9 min/sample
Triglycerides	9 min/sample
Dengue rapid	10 min/sample
Hepatitis B antigen rapid	10 min/sample
Hepatitis B (AHBS)—automated	9 min/sample
Hepatitis BHBc—automated	9 min/sample
Hepatitis B IgG	9 min/sample
HIV—automated	29 min/sample
HIV rapid	31 min/sample
Rapid syphilis	10 min/sample
Pap smear staining	33 min/sample
Acid fast bacilli	29 min/sample
Urinalysis—automated	4 min/sample
Urinalysis—manual	8 min/sample
Faecalysis	23 min/sample

Workload Group 2: Support Activities	Activity Standard
Internal quality control (IQC)	30 min/day
Calibration of laboratory equipment	20 min/day
Inventory management	1 h/month
Technical Evaluation of New Equipment	3 h/year
Validation of test parameters	8 h/year
Advocacy lecture	2 h/week
Mobile blood collection	6 h/year
External quality control	1 h/month
Departmental meetings	2 h/month
Continuous professional development	5 days/year

Workload Group 3: Additional Activities	Activity standard Number of staff per additional activity varied per facility
Registration of health certificates	1 h/day
Management meetings and review	4 h/month
Supervision of staff	30 min/day
Orientation new staff	2 h/year
Documentary requirements for License to Operate	30 min/year
Monthly reports	1 h/month
Billing forms	30 min/month
Quality manual review	2 h/year
Research	2 h/month

**Table 3:** Workload components and activity for physicians

Workload Group 1: Health Service Activities	Activity standard
Consultations	16 min/patient
Minor surgical procedures	30 min/patient
Referrals	9 min/patient
Family planning—bilateral tubal ligation (BTL)	30 min/patient
Family planning—vasectomy	30 min/patient

Workload Group 2: Support Activities	Activity standard
Workload Group 2: Support Activities	Activity standard
Health education	2 h/month
Departmental meetings	2 h/month
Continuing professional development	8 days/year
Outreach program (medical mission)	8 h/month
Issuance of documents and medicolegal management	8 h/month

Workload Group 3: Additional Activities	Activity standard Number of staff per additional activity varied per facility
Staff supervision	30 min/day
Trainee supervision	2 h/week
Administrative functions	1 h/week
Interpretation and action on surveillance	2 h/week

**Table 4:** Workload components and standards for midwives

Workload Group 1: Health Service Activities	Activity standard
Antenatal visits	39 min/client
Family planning—male condoms	24 min/client
Family planning—injectables	23 min/client
Family planning—intrauterine device (IUD)	53 min/client
Family planning—natural	33 min/client
Family planning—implants	44 min/client
Family planning—pills	12 min/client
Normal spontaneous delivery	99 min/patient
Newborn care	120 min/patient
Labor management	168 min/patient
Post-natal care	35 min/patient
Childcare/well baby clinic	18 min/patient
Integrated management of childhood illness	22 min/patient
Visual inspection with acetic acid (VIA)	25 min/patient
Pap smear	20 min/patient
Rehabilitation of malnourished children	20 min/patient
Caesarean section (pre-operative care)	14 min/patient

Workload Group 2: Support Activities	Activity standard
Health education	30 min/day
Home visits	8 h/month
Staff meetings	2 h/month
Continuous professional development	48 h/year
Medical missions	16 h/month
Housekeeping (5S) practice	40 min/day
Mentoring of students	12 h/week

Workload Group 3: Additional Activities	Activity standard Number of staff per additional activity varied per facility
Supervision of BHWs	I hour/day
Management meetings	I hour/month
Mass circumcision	8 h/year

**Table 5:** Workload components and standards for nurses

Workload Group 1: Health Service Activities	Activity standard
Patient assessment	13 min/patient
Nursing diagnosis and management	34 min/patient
Minor surgical procedures	37 min/patient
Wound care	30 min/patient
Administration of medication	20 min/patient
Immunization	12 min/patient
External referral with escort	142 min/patient
Internal referral/external referral without escoWorkload Group 1: Health Service Activities rt	12 min/patient

Workload Group 2: Support Activities	Activity standard
Health teachings	30 min/day
Home visits	8 h/week
Reporting patient census	30 min/day
Staffing meetings	1 h/month
Community outreach programs	8 h/month
Group counselling	2 h/month
Continuing education program	2 h/month

Workload Group 3: Additional Activities	Activity standard Number of staff per additional activity varied per facility
Disease Surveillance	1 h/month
Supervision of staff	1 h/day
Staff scheduling	1 h/week
Mentoring of students	1 h/week
Management meetings	2 h/month
Supervisor’s monthly reports	1 h/month
Performance evaluation	2 h/year
Nursing audit	2 h/month
Committee work	3 h/month

### Staffing requirements calculated

The 2018 annual service statistics were collected from the BHSs and RHUs/CHOs uploaded and analysed using the WISN software to determine staffing requirements for the specific facilities. The results were provided as differences and ratios. Table 6 provides a summary of computed required staffing per cadre in each of the BHS with midwives. The differences show shortages, excesses, and balances, while the ratios show the levels of workload pressure whether high or low.

**Table 6:** WISN calculated staffing requirements for midwives in barangay health stations

Facility	Existing staff	WISN calculated staff	Difference	WISN ratio
BHS_1_	1	2	− 1	0.50
BHS_2_	2	1	1	2.00
BHS _3_	1	1	0	1.00
BHS _4_	1	1	0	1.00
BHS _5_	1	2	− 1	0.50
BHS _6_	1	2	− 1	0.50
BHS _7_	2	1	1	2.00
BHS _8_	1	1	0	1.00
BHS _9_	2	2	0	1.00
BHS _10_	2	2	0	1.00
BHS _11_	1	1	0	1.00
BHS _12_	3	3	0	1.00
BHS _13_	1	1	0	1.00
BHS _14_	1	1	0	1.00
BHS_15_	1	1	0	1.00
BHS_16_	1	2	− 1	0.50
BHS_17_	1	2	− 1	0.50
BHS_18_	1	2	− 1	0.50
BHS_19_	2	2	0	1.00
BHS_20_	2	2	0	1.00
BHS_21_	2	1	1	2.00
BHS _22_	2	1	1	2.00
BHS _23_	2	2	0	1.00
BHS _24_	2	2	0	1.00
BHS_25_	5	2	3	2.50
BHS_26_	2	1	1	2.00
BHS_27_	2	1	1	2.00
BHS_28_	2	1	1	2.00
BHS_29_	2	1	1	2.00
BHS_30_	2	1	1	2.00
BHS_31_	2	1	1	2.00
BHS_32_	1	1	0	1.00
BHS_33_	1	1	0	1.00
BHS_34_	1	1	0	1.00
BHS_35_	1	1	0	1.00
BHS_36_	1	1	0	1.00
BHS_37_	1	1	0	1.00
BHS_38_	2	1	1	2.00
BHS_39_	1	2	− 1	0.50
BHS_40_	1	1	0	1.00
BHS_41_	1	1	0	1.00
BHS_42_	2	2	0	1.00
BHS_43_	1	1	0	1.00
BHS_44_	2	1	1	2.00
BHS_45_	1	1	0	1.00
BHS_46_	1	1	0	1.00
BHS_47_	2	1	1	2.00
BHS_48_	1	1	0	1.00
BHS_49_	1	2	− 1	0.50

Most BHS have only midwives supervising BHWs who are not part of the professionalized workforce

At the BHSs, results on staffing differed across the cadres. Some facilities exhibited shortages, others balance between existing staff and calculated needs, while some facilities exhibited surpluses. The midwives did not perform the full range of services for the level of care as provided for in the BHS package of services. Out of the 49 BHS, eight (16%) recorded shortages, 24 (49%) operated normally with enough staff, while 17 (34%) of the Barangays Health Stations registered staff surpluses.

Further results on staffing requirements for the RHUs/CHOs are provided in the annex in Table 7 annexed. Six (15%) of the RHUs/CHOs registered staffing shortages, none operated at normal levels with sufficient staff and 34 (85%) health facilities recorded staff surpluses. A total of 10 RHUs/CHOs in the study had no medical technologists.

**Table 7:** WISN Results for the Four Cadres in RHUs/CHOs

Facility	Cadre	Existing staff	WISN calculated staff	Difference	WISN ratio
RHU_1_	Med. Tech	1	1	0	1.00
	Physician	1	1	0	1.00
	Nurse(OP)	1	1	0	1.00
RHU_2_	Med. Tech	1	1	0	1.00
	Physician	1	1	0	1.00
	Nurse(OP)	1	2	− 1	0.50
	Midwife	1	1	0	1.00
RHU_3_	Med. Tech	2	1	1	2.00
	Physician	1	2	− 1	0.50
	Nurse(OP)	3	4	− 1	0.75
RHU_4_	Med. Tech	1	1	0	1.00
	Physician	1	1	0	1.00
	Nurse(OP)	3	1	2	3.00
RHU_5_	Med. Tech	3	2	1	1.50
	Physician	2	8	− 6	0.25
	Midwife	5	3	2	1.67
	Nurse(OP)	5	8	− 3	0.63
RHU_6_	Med. Tech	1	1	0	1.00
	Physician	1	2	− 1	0.50
	Midwife	3	2	1	1.50
	Nurse(OP)	4	1	3	4.00
RHU_7_	Med. Tech	1	1	0	1.00
	Physician	1	1	0	1.00
	Midwife	12	3	9	4.00
	Nurse(OP)	8	2	6	4.00
RHU_8_	Med. Tech	1	1	0	1.00
	Physician	1	2	− 1	0.50
	Midwife	2	2	0	1.00
	Nurse(OP)	2	3	− 1	0.67
RHU_9_	Med. Tech	3	2	1	1.50
	Physician	4	2	2	2.00
	Midwife	7	3	4	2.33
	Nurse(OP)	6	7	− 1	0.86
RHU_10_	Med. Tech	1	1	0	1.00
	Physician	1	5	− 4	0.20
	Midwife	1	1	0	1.00
	Nurse(OP)	3	6	− 3	0.50
RHU_11_	Med. Tech	1	1	0	1.00
	Physician	3	4	− 1	0.75
	Midwife	8	5	3	1.60
	Nurse(OP)	5	8	− 3	0.63
RHU_12_	Med. Tech	2	1	1	2.00
	Physician	2	2	0	1.00
	Midwife	14	4	10	3.50
RHU_13_	Med. Tech	1	1	0	1.00
	Physician	1	1	0	1.00
	Midwife	8	1	7	8.00
	Nurse(OP)	2	2	0	1.00
RHU_14_	Med. Tech	2	3	− 1	0.67
	Nurse(OP)	14	3	11	4.67
RHU_15_	Med. Tech	1	1	0	1.00
	Physician	1	3	− 2	0.33
	Midwife	8	5	3	1.60
	Nurse(OP)	7	1	6	7.00
RHU_16_	Physician	1	1	0	1.00
	Midwife	3	2	1	1.50
	Nurse(OP)	13	2	11	6.50
RHU_17_	Midwife	4	2	2	2.00
RHU_18_	Nurse(OP)	2	2	0	1.00
RHU_19_	Med. Tech	3	1	2	3.00
	Physician	1	1	0	1.00
	Midwife	3	2	1	1.50
	Nurse(OP)	1	1	0	1.00
RHU_20_	Physician	1	1	0	1.00
	Midwife	4	1	3	4.00
	Nurse(OP)	9	2	7	4.50
RHU_21_	Med. Tech	1	1	0	1.00
	Physician	1	1	0	1.00
	Midwife	7	6	1	1.17
	Nurse(OP)	8	4	4	2.00
RHU_22_	Med. Tech	1	1	0	1.00
	Physician	1	2	− 1	0.50
	Midwife	6	4	2	1.50
	Nurse(OP)	5	2	3	2.50
RHU_23_	Midwife	1	1	0	1.00
	Nurse(OP)	1	1	0	1.00
RHU_24_	Physician	1	2	− 1	0.50
	Midwife	1	3	− 2	0.33
	Nurse(OP)	1	4	− 3	0.25
RHU_25_	Physician	1	2	− 1	0.50
	Midwife	8	5	3	1.60
	Nurse(OP)	1	4	− 3	0.25
RHU_26_	Physician	1	2	− 1	0.50
	Midwife	1	5	− 4	0.20
	Nurse(OP)	1	5	− 4	0.20
RHU_27_	Med. Tech	2	2	0	1.00
	Physician	1	2	− 1	0.50
	Midwife	16	12	4	1.33
	Nurse(OP)	13	4	9	3.25
RHU_28_	Med. Tech	3	6	− 3	0.50
	Physician	3	4	− 1	0.75
	Nurse(OP)	6	5	1	1.20
RHU_29_	Med. Tech	3	1	2	3.00
	Physician	1	4	− 3	0.25
	Nurse(OP)	3	4	− 1	0.75
RHU_30_	Med. Tech	3	3	0	1.00
	Physician	1	5	− 4	0.20
	Nurse(OP)	5	2	3	2.50
RHU_31_	Med. Tech	2	3	− 1	0.67
	Physician	2	4	− 2	0.50
	Nurse(OP)	3	2	1	1.50
RHU_32_	Med. Tech	1	2	− 1	0.50
	Physician	5	6	− 1	0.83
	Nurse(OP)	3	5	− 2	0.60
RHU_33_	Med. Tech	1	1	0	1.00
	Physician	1	2	− 1	0.50
	Midwife	18	5	13	3.60
	Nurse(OP)	1	1	0	1.00
RHU_34_	Midwife	24	4	20.06	6.09
RHU_35_	Med. Tech	2	3	− 1	0.67
	Physician	1	2	− 1	0.50
	Midwife	5	1	4	5.00
	Nurse(OP)	3	3	0	1.00
RHU_36_	Med. Tech	1	1	0	1.00
	Physician	2	3	− 1	0.67
	Midwife	5	7	− 2	0.71
	Nurse(OP)	2	2	0	1.00
RHU_37_	Physician	1	3	− 2	0.33
	Nurse(OP)	3	2	1	1.50
RHU_38_	Physician	1	1	0	1.00
	Nurse(OP)	2	1	1	2.00
RHU_39_	Med. Tech	1	3	− 2	0.33
	Physician	1	2	− 1	0.50
	Midwife	8	5	3	1.60
	Nurse(OP)	2	2	0	1.00
RHU_40_	Med. Tech	1	1	0	1.00
	Physician	1	1	0	1.00
	Nurse(OP)	5	2	3	2.50

### Workload pressure

**Fig. 1:**
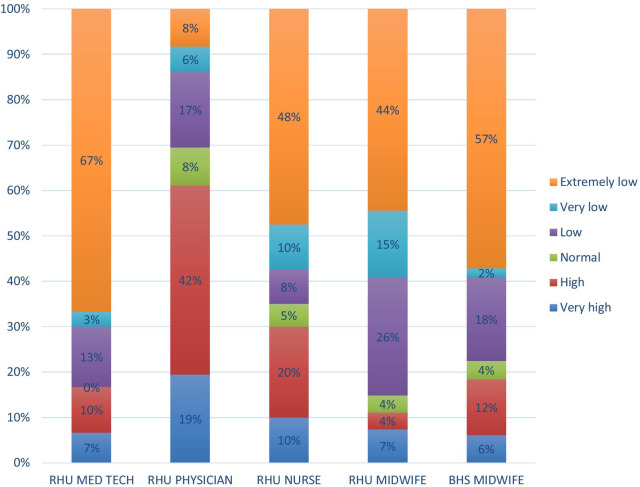
Summary of workload pressure by cadre and facility

The workload pressure for each of the cadres was examined (see Fig. 1). Using the WISN difference and ratio, a staff difference 0 and ratio of 1.00 was rated as having normal workload pressure. In circumstances, where staff required showed a difference of -1 and ratio of 0.50, the workload pressure was rated as high and where the staff difference was less by 2 and above with a WISN ratio of -2.00, the workload pressure was rated as very high. Where staff calculated was more than the required by 1, 2 3 and above, workload pressures ranged between low, very low and extremely low, respectively. In all the 49 Barangay health stations, 57% of the midwives functioned at extremely low workload pressures. On the other hand, 20% exhibited low and very low workload pressures, 18% experienced high and very high workload pressures, while only 4% of the midwives operated at normal workload pressures.

At the RHUs, the situation was similar with midwives recording extremely low workload pressure at 44%, followed by nurses at 48% and medical technologists at 67% Physicians registered 8% extremely low workload pressures.

### Workload analysis across all workload groups

**Fig. 2:**
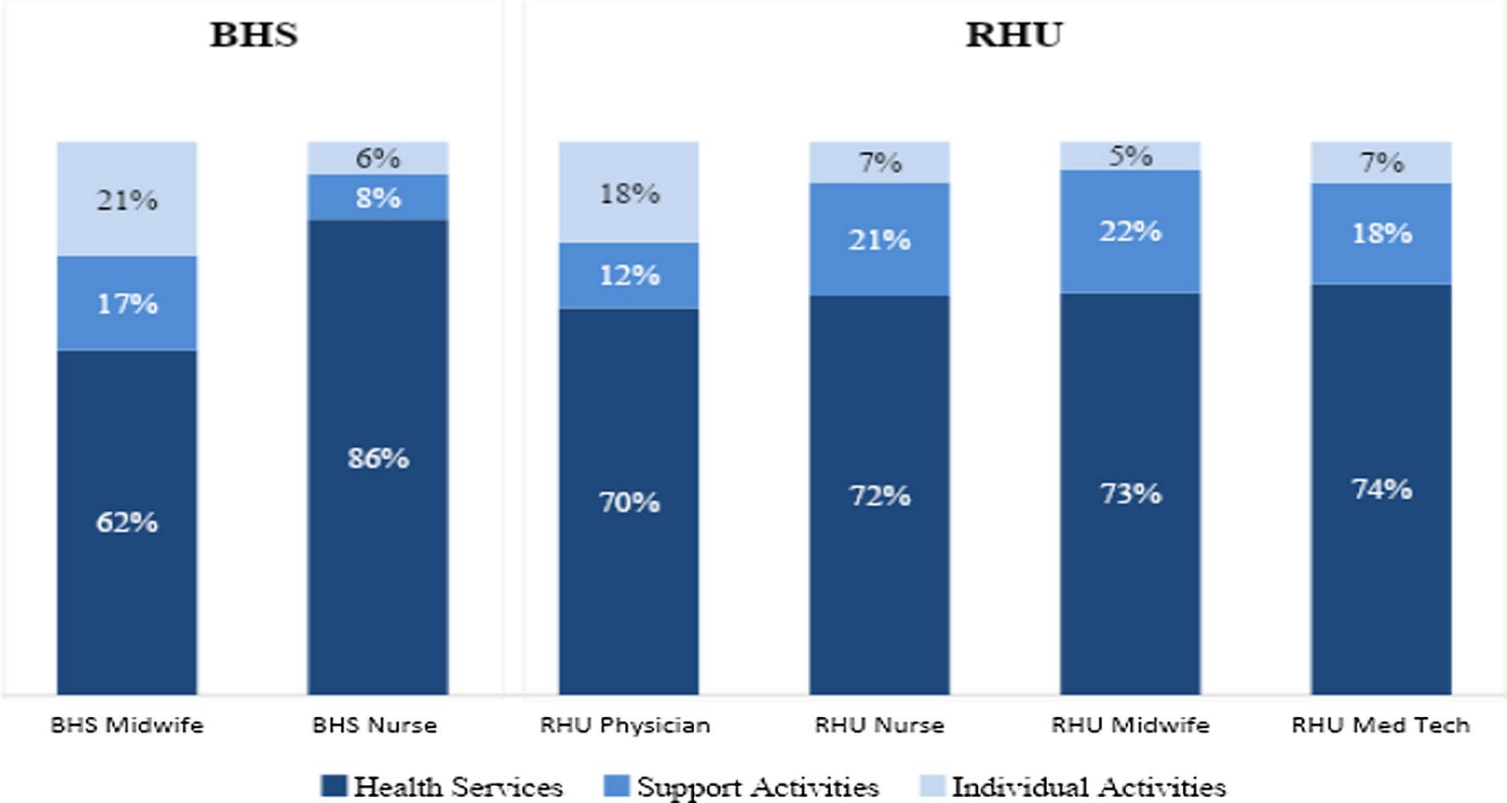
Summary of workload analysis by cadre and facility

The study also looked at how the health workers spent their AWT across health service activities, support and additional activities (see Fig. 2). The findings show that RHU medical technologists, midwives and nurses spend about 20% of time in support activities, with physicians spending 18% of their time on individual activities. Midwives at BHS also spent nearly 38% of their time on support and individual activities, leaving only 62% for health services, in contrast to the Nurses 86% of time spent on health services.

Results demonstrated many trends in provision of services which impacted workload. There were differences in services offered at the same levels of care, or PHC services being referred to level one hospital, despite the Philippines having a prescribed package of health services for each level of care. The study also found that some health workers were regularly undertaking tasks that were not a part of their scopes of practice. In addition, overlapping and shifting of tasks among the health workers was seen. For example, FP services and immunizations were offered by the nurses, midwives or even the physicians. BHWs are also involved in offering selected FP services an indication of tasks being shared and shifted. In addition, results revealed that cadres, such as medical technologists hired by partners for TB programs only conducted TB diagnosis related activities.

## Discussion

Findings from this WISN study highlight the health workforce situation at the primary care facilities. The study results show that the health workers AWT differed for each of the cadres at the various facilities. For example, midwives spent more time in BHSs due to fewer days taken for leave, training, or other absences. It emerged that there were inequitable days for training for each of the cadres with the physicians having more days for training compared to their other colleagues. The lack of a coordinated approach and weak supervision and management of health worker training at the LGUs is likely to exacerbate HRH absences thus affecting access to services. While continuous training to update health worker skills is critical for both motivation and improving effectiveness it should be well distributed within the cadres to enhance productivity [22]. This finding supports rationale for ongoing efforts to apply innovative training approaches, such as use of eLearning to enhance competency of staff with less disruption in health service delivery.

The differences in services offered at the various BHSs and RHUs demonstrate that facilities are not offering the prescribed standard package of services. Midwives mainly provided services, such as consultations for minor illnesses, selected maternal and child health services, such as FP, antenatal, postnatal care, and immunizations. Midwives have the clinical skills and scope of practice to undertake more services, but it was found they do not offer all services described by the BHS package of health services. Similarly, the medical technologists offered very minimal services, focusing mainly on acid-fast bacilli testing, urinalysis and faecalysis, from a wide range of services they can offer. This finding provides evidence for the revision of the existing standard packages of services for health facilities to match the needs of communities. In other countries, WISN studies have been used to revise the services offered at various levels of care in other countries [23, 24]. As such, the WISN study recommended to the Steering Committee that an update of the standard package of services for PHC was needed. By 2020, during the drafting of this manuscript, the DOH used the study results to inform development of the essential package of services for primary care facilities including staffing norms considering the WISN results.

High and extremely high workloads and pressure recorded in some facilities indicate staff shortages that may compromise quality of services provided. Other WISN studies have equally reported high pressure at various levels and cadres and recommended staff redistribution of health workers to facilities with high workloads [25]. Recognizing that maximizing the potential of the health workforce is one of the policy orientations specified in the UHC law, the WISN results provided evidence that resulted in defining staffing minimum and maximum requirements at primary care facilities as shown in Table 8. The study found that a BHS requires a minimum of one midwife and maximum of two midwives as well as a minimum of two nurses and a maximum of three nurses to offer the wide range of services at these units. Similarly, RHUs/CHOs require a minimum of one and a maximum of two laboratory technologists. The RHUs/CHOs also require two nurses and two midwives at the minimum and a maximum four nurses and midwives, respectively. Finally, RHUs/CHOs require a minimum of one and two maximum physicians.

**Table 8:** Proposed staffing levels as defined by WISN method for primary care facilities

BHS		
Health worker type	Average of minimum staffing requirement	Average of maximum staffing requirement
Midwife	1	2
Nurse	2	3

RHU		
Health worker type	Average of minimum staffing requirement	Average of maximum staffing requirement
Physician	1	2
Nurse	2	4
Midwife	2	4
Med Tech	1	2

Such results have been reported in WISN studies conducted in other countries that have expanded services in the community units as part of primary care [26–28]. Globally, countries are increasingly turning to community health workers, or BHWs in the Philippines, to extend health services to underserved areas [29]. During the study interviews, the researchers found that BHWs contributed to most of the workloads on PHC services captured in BHSs and some RHUs. Supporting and recognizing the importance of BHWs, through relevant short training, supervision, and provision of equipment, is a critical opportunity to achieve UHC [30].

### Key policy changes recommended by the study

Based on the results, the following recommendations were proposed:

1. **Review of service packages at the primary care levels.** The results recorded different services at the same levels of facilities despite the existence of a prescribed service delivery model with key services to be offered. In addition, some facilities rated themselves differently compared to the rating in the master facility list of the country. There is a need to reclassify facilities according to their capacities at various levels of the health system. Finally, to guide the catchment population, lists of services under the mandate of the facility to provide should be published publicly.
2. **Redistribution of staffing based on the workloads for particular facilities.** There were instances, where some facilities had more staff with less workload, while others had high workloads with less staff within the province, LGU and facility levels. Staff redistribution using population health trends from highly staffed facilities within the LGUs and provinces under the same jurisdiction or health care provider network can be a rational approach to balance workloads.
3. **Review of implications of donor funded programs on staffing.** It was noted that the medical technologists hired by partners for TB programmes only conducted TB related activities. Because of this, clients who needed tests for various conditions were referred to other facilities. This is not a sustainable practice given funding cycles of projects, results in underutilization of trained health professionals, and limits accessibility to key health services. This practice should be examined, and WISN results should be used to guide decision making regarding all staff deployed in the facilities regardless of the employer.
4. **Revision of scopes of practice and job descriptions.** It was found that most cadres informally undertake tasks that originally are not part of their training or tasks that have been shifted from another cadre. This calls for revision of scopes of practice and creation of new cadres, such as those at the assistant level. In addition, a task shifting and task sharing policy should be developed to formalize the shifting and sharing practices already being undertaken, ensuring adequate training and supervision are emphasised.
5. **Development of referral guidelines.** There were instances, where referrals were made to a cadre of the same competency at a higher level of care. The study found that often services meant to be conducted at the BHSs or RHUs were referred to level one hospitals. To curb unnecessary referrals to the next level of care, referral guidelines should be developed.
6. **Improvement in data collection and investing in health information systems.** The need for improved data collection and record keeping was emphasised. Although many of the facilities used the same tools, the modes of reporting differed and data on some key indicators were missing in the final reporting tool. The DOH and health facilities should invest in strengthening collection and storage systems for health services and HRH data, as well as linking these systems through an interoperable health management information system to ensure availability of quality data in real time.
7. **Strengthening supervision and management of resources.** The study found there was a need to strengthen the leadership capacities of the health workers who had additional activities related supervision of resources. Without these skills, it is difficult for the health workers tasked with the roles to supervise and manage the resources (staff, financial, equipment and infrastructure) effectively. Use of non-face-to-face modalities for training may be optimized to increase available working time and lessen disruption in health services. Supportive supervision of staff can also promote efficiency gains by minimizing time allotted for repetitive training.

### Best practices for countries wanting to implement and integrate WISN into HRH management practices

In the Philippines, WISN was not implemented as a one-time activity, but implemented with the intention of long-term integration into HRH management. Several best practices were identified to facilitate this integration:

1. Development of a governance structure that includes representation of the high-level officials and members of the DOH both for a regular standing committee and for the steering committee,
2. Having a dedicated core team from the HRH department, who conduct all planning, lead implementation of WISN, analysis of results, and work closely with the Regional Health Offices.
3. Development of a sustainability strategy and plan to guide needed policy modifications to reflect the needs and outputs of WISN, promote domestic resource mobilization to carry out the approach and overall planning for application of results.
4. Creating a culture of continuous learning. For example, the Philippines developed an online WISN orientation course for all HR management officers and members of WISN committees, new and old, as part of continuous professional development through an eLearning platform.
5. Development of a basic toolkit for training, carrying out WISN and analysing results, relevant and unique to the country.

## Conclusion

There is a need for the Philippine health system, both through the DOH and the local government to efficiently optimize the available health workers for PHC. The results provide evidence for staffing requirements in the primary care facilities by defining the minimum and maximum numbers based on workloads, scope of practice and time taken to undertake specific tasks at the barangay health stations, rural health units and city health offices. WISN results can be integrated into the human resources for health management systems.

## Data Availability

Data is available upon request to the Health Human Resources Development Bureau at psd.hhrdb@doh.gov.ph.

## Abbreviations

AWT: Available Working Time
BHS: Barangay Health Station
BHW: Barangay Health Worker
CHO: City Health Office
DOH: Department of Health
EWG: Expert Working Group
HHRDB: Health Human Resources Development Bureau
HRH: Human Resources for Health
HRH2030: Human Resources for Health in 2030 Program
HRO: Human Resource Officer
IRR: Implementing Rules and Regulations
LGU: Local Government Units
OP: Outpatient
RHU: Rural Health Unit
TTF: Technical Task Force
UHC: Universal Health Care
USAID: United States Agency for International Development
WISN: Workload Indicators of Staffing Need
WHO: World Health Organization
